# Dr. Emile De Trey

**Published:** 1898-11

**Authors:** 


### DR. EMILE DE TREY.

Dr. de Trey died at Zurich, Switzerland, August 3, 1898. He was born at Vevay, Switzerland, in 1846. He began the study of dentistry, at twenty years of age, at the University of Berne, where he remained for one year. He came to the United States in 1867, and entered the Pennsylvania College of Dental Surgery, graduating from that institution, and returned to his native country, continuing his studies at the University of Berne, from which he received his diploma in 1869. He began practice in Vevay, and continued there for twenty-three years, and subsequently removed to Basel, where he remained in practice to the close of his life.

He was one of the founders of the American Dental Society of Europe, and also a member of the Swiss Odontological Society.

His introduction of the peculiar form of sponge gold, known throughout the dental profession as the "solila gold," made his name a household word wherever dentistry was taught and practised.

The writer knew him as a student of marked ability, and during all of his subsequent life has observed with increasing interest his earnestness and devotion to the good of his profession. His ability as a practitioner was of a high order, and throughout his professional life he maintained an elevated standard, his career continuing an honor to the school from which he received his degree. His comparatively early departure from the active duties of life is a personal as well as a professional loss.

The sympathy of those who appreciated his life and work on this continent is extended to his family in their severe bereavement, and to our *confrères* in Europe for the loss of a colleague worthy their honor and respect.

Dr. de Trey married Mlle. Leah Pittet, of Vevay. Six sons and two daughters survive him.

